# Exposure to Family Member Incarceration and Adult Well-being in the United States

**DOI:** 10.1001/jamanetworkopen.2021.11821

**Published:** 2021-05-28

**Authors:** Ram Sundaresh, Youngmin Yi, Tyler D. Harvey, Brita Roy, Carley Riley, Hedwig Lee, Christopher Wildeman, Emily A. Wang

**Affiliations:** 1 Department of Internal Medicine, University of California, Los Angeles; 2 Department of Sociology, University of Massachusetts, Amherst; 3 SEICHE Center for Health and Justice, Yale School of Medicine, New Haven, Connecticut; 4 Department of Internal Medicine, Yale School of Medicine, New Haven, Connecticut; 5 Department of Chronic Disease Epidemiology, Yale School of Public Health, New Haven, Connecticut; 6 Department of Pediatrics, University of Cincinnati College of Medicine, Cincinnati, Ohio; 7 Division of Critical Care, Cincinnati Children’s Hospital Medical Center, Cincinnati, Ohio; 8 Department of Sociology, Washington University in St Louis, St Louis, Missouri; 9 Department of Sociology, Duke University, Durham, North Carolina; 10 Rockwool Foundation, Copenhagen, Denmark

## Abstract

**Importance:**

More than half of the adult population in the United States has ever had a family member incarcerated, an experience more common among Black individuals. The impacts of family incarceration on well-being are not fully understood.

**Objective:**

To assess the associations of incarceration of a family member with perceived well-being and differences in projected life expectancy.

**Design, Setting, and Participants:**

This nationally representative cross-sectional study used data from the 2018 Family History of Incarceration Survey to examine how experiences of family member incarceration were associated with a holistic measure of well-being, including physical, mental, social, financial, and spiritual domains. Well-being was used to estimate change in life expectancy and was compared across varying levels of exposure to immediate and extended family member incarceration using logistic regression models to adjust for individual and household characteristics. Data were analyzed from October 2019 to April 2020.

**Exposures:**

Respondents’ history of family member incarceration, including immediate and extended family members.

**Main Outcomes and Measures:**

The main outcome was self-reported life-evaluation, a measure of overall well-being from the 100 Million Healthier Lives Adult Well-being Assessment. Respondents were considered thriving with a current life satisfaction score of 7 or greater and a future life optimism score of 8 or greater, each on a scale of 0 to 10. Other outcomes included physical health, mental health, social support, financial well-being, and spiritual well-being, each measured with separate scales. Additionally, life expectancy projections were estimated using population-level correlations with the Life Evaluation Index. All percentages were weighted to more closely represent the US population.

**Results:**

Of 2815 individuals included in analysis, 1472 (51.7%) were women, 1765 (62.8%) were non-Hispanic White, and 868 (31.5%) were aged 35 to 54 years. A total of 1806 respondents (45.0%) reported having an immediate family member who was incarcerated. Compared with respondents with no family incarceration, any family member incarceration was associated with lower well-being overall (thriving: 69.5% [95% CI, 65.0%-75.0%] vs 56.9% [95% CI, 53.9%-59.9%]) and in every individual domain (eg, physical thriving: 51.1% [95% CI, 46.2-56.0] vs 35.5% [95% CI, 32.6%-38.3%]) and with a mean (SE) estimated 2.6 (0.03) years shorter life expectancy. Among those with any family incarceration, Black respondents had a mean (SE) estimated 0.46 (0.04) fewer years of life expectancy compared with White respondents.

**Conclusions and Relevance:**

These findings suggest that family member health and well-being may be an important avenue through which incarceration is associated with racial disparities in health and mortality. Decarceration efforts may improve population-level well-being and life expectancy by minimizing detrimental outcomes associated with incarceration among nonincarcerated family members.

## Key Points

**Question:**

Is the incarceration of a family member associated with well-being and projected life expectancy?

**Findings:**

In this cross-sectional study including 2815 individuals, any family member incarceration was associated with lower well-being and a projected 2.6-year reduction in life expectancy compared with no family member incarceration experience. Among those with any family incarceration, Black respondents had an estimated 0.5 fewer years of projected life expectancy compared with White respondents.

**Meaning:**

These findings suggest that efforts to decarcerate may improve population-level health and well-being by reducing racial disparities and detrimental outcomes associated with incarceration for nonincarcerated family members.

## Teaser

This cross-sectional study assesses the associations of incarceration of a family member with perceived well-being and projected life expectancy among adults in the US.

## Introduction

The last 4 decades have been marked by unprecedented levels of incarceration in the United States, especially in Black communities. An estimated 63% of Black individuals in the US have had an immediate family member who was incarcerated, compared with 45% of individuals in the US overall.^1^

A sizeable body of evidence has illustrated the impacts of incarceration on particular facets of family life of incarcerated individuals.^2,3,4^ Having a family member incarcerated can damage the economic stability of already financially tenuous families, who are then at greater risk of criminal legal system involvement.^3,5^ Individuals with family members who are incarcerated are more likely to have reduced social support owing to community stigma attached to incarceration.^6,7^ This reduced social support spans generations, with children and grandparents experiencing stigmatization and financial consequences and grandparents experiencing caregiver burden.^7,8^

In addition to outcomes in family social dynamics, incarceration is associated with negative long-term health consequences for family members.^2,7,8,9^ Women with partners who are incarcerated are more likely to experience depression, hypertension, and diabetes.^10^ Children with parents who are incarcerated are at greater risk of having worse mental health, substance use, and obesity, with potential long-term risks into adulthood.^7,11^ Although there is limited research examining the intergenerational health outcomes associated with incarceration, there is evidence that grandparents with adult children who are incarcerated experience greater psychological distress and depression, especially those with caregiving roles.^12,13^

These data suggest that incarceration of a family member is associated with an impact on broader well-being, defined as a holistic condition encompassing physical health, as well as emotional, social, and spiritual components. In 1948, the World Health Organization defined health as “a state of complete physical, mental and social well-being and not merely the absence of disease or infirmity.”^14^ Aligned with this definition, well-being extends beyond the presence or absence of specific physical health outcomes and assesses broader lived experiences, such as overall life satisfaction or experiences of overall suffering or thriving.^15^ Population-level well-being is associated with key indicators of population health, such as life expectancy,^16^ and can sensitively reflect societal contextual factors, like social stability, political freedom, and economic security.^17^

Although prior studies have separately examined social, economic, and health outcomes associated with family member incarceration, no prior literature has examined how the incarceration of a family member is associated with a comprehensive measure of well-being using a national population based study, to our knowledge. To fill this gap, we aimed to explore the association between family member incarceration and well-being.

## Methods

This cross-sectional study was classified as exempt from review or further informed consent by the institutional review board at the Yale School of Medicine because it used deidentified, publicly available data for a secondary data analysis. We followed the Strengthening the Reporting of Observational Studies in Epidemiology (STROBE) reporting guideline for cross-sectional studies.

### Study Sample

We used data from the Family History of Incarceration Survey (FamHIS), a nationally representative cross-sectional study about family incarceration experiences in the United States.^1^ FamHIS investigators worked with the National Opinion Research Center to recruit a baseline sample of 4041 participants and collect data between August and September 2018. The baseline sample was derived from the National Opinion Research Center AmeriSpeak sampling frame and was designed to be nationally representative of the US household adult population via stratified sampling by age, self-identified gender, race/ethnicity, and education. Oversampling in the sample design and sample weighting in the analysis were used to account for lower participation rates among young adults and Black and Hispanic individuals, and both internet- and telephone-based surveys were provided to include hard-to-reach populations.

Participants in this baseline sample completed a brief screening tool that assessed exposure to immediate family member incarceration. Using prespecified target sample sizes to accurately estimate the prevalence of family member incarceration, the National Opinion Research Center invited screened participants who experienced any immediate family member incarceration and a random subset of individuals who did not experience any immediate family incarceration to complete the full questionnaire, which included items on well-being, individual exposure to incarceration, and family member exposure to incarceration. This yielded a total of 1806 respondents with immediate family member incarceration experience and 1009 respondents without immediate family member incarceration experience in the full study sample. The survey completion rate was 69.7% of those who were initially screened, which closely aligns with the original target sample sizes. Details on the sampling design and weighting methods are listed in eAppendix 1 in the Supplement and in prior work.^1^

### Dependent Variable

Our main outcome was self-reported life evaluation, a measure of overall well-being based on the Cantril Self-Anchoring Striving Scale,^18^ which has been used extensively in research on well-being in the US and internationally.^18^ Participants ranked current life satisfaction and future life optimism on scales from 0 to 10, using an image of a ladder to help visualize and conceptualize the scale (eFigure in the Supplement). Responses of current life satisfaction of 7 or greater and future life optimism of 8 or greater were classified as a thriving life evaluation. Responses of current life satisfaction and future life optimism of 4 or less were classified as a suffering life evaluation; all other combinations of responses were classified as a surviving life evaluation.^15^ Estimates of life expectancy were based on the Life Evaluation Index, calculated for any population subset as (*%Thriving* − *%Suffering*) × 100. An increase of 1 SD in the Life Evaluation Index (mean [SD], 48 [5.4]) is estimated to be associated with a 1.54-year longer life expectancy at the population level, as derived from county-level data from previous work.^16^

Well-being was assessed using the 100 Million Healthier Lives Adult Well-being Assessment,^15^ which includes a set of reliable and validated quantitative scales that measure well-being overall and by specific domains.^19,20,21,22^ Other well-being domains included physical health, mental health, social support, spiritual well-being, and financial well-being. Physical health, mental health, and social support were self-rated on 5-point Likert scales. Spiritual well-being was measured using a 7-point Likert scale that evaluated respondents’ sense of purpose and life meaning. Financial well-being was measured using an 11-rung ladder similar to the Cantril self-anchoring scale (eAppendix 2 in the Supplement). Responses for each domain of well-being were categorized using 100 Million Healthier Lives–specified cutoffs for each scale (eTable 1 in the Supplement).

### Covariates

We measured respondent age, gender, race/ethnicity, education level, income, housing type, employment status, marital status, family size, and self-reported history of drug or alcohol addiction and incarceration history. Age was categorized as 18 to 24 years, 35 to 44 years, 45 to 54 years, 55 to 64 years, 65 to 74 years, and 75 years and older. Household income was categorized by quintiles based on 2012 to 2016 American Community Survey.^1^ We conceptualize self-identified race as an important indirect proxy of structural racism, and to avoid the quantitative erasure^23^ of this crucial aspect of incarceration, we used a 2-pronged analytic strategy: we included race/ethnicity as a covariate in regression models to identify an adjusted main association between family member incarceration exposure and well-being, and we calculated race-stratified estimates of key statistics to explore differences in well-being associations with family member incarceration exposure by race.

### Independent Variable

The independent variable was respondents’ history of family member incarceration. Respondents were asked if they had any immediate family members who had ever been incarcerated, defined as parents, partners (ie, married spouses, unmarried romantic partners, and coparents of children), siblings, or children. Respondents were then asked the total number of immediate family members and number of incarcerated family members in each relationship type (eg, number of siblings) and the longest duration for which any of their immediate family members had been incarcerated (categorized as 1 day, 2 days to 1 month, >1 month to 1 year, >1 to 5 years, 6-10 years, or >10 years).

Respondents were asked if they had any extended family members who had ever been incarcerated, including grandparents, aunts and uncles, nieces and nephews, cousins, godparents, mothers- or fathers-in-law, sisters- or brothers-in-law, or any other nonimmediate family member. We used a dichotomous measure of any family incarceration to identify respondents who reported having any immediate or extended family member who had ever been incarcerated.

### Statistical Analysis

We examined the demographic and socioeconomic characteristics of respondents based on family member incarceration exposure and used χ^2^ and Wilcoxon rank-sum tests to assess differences in these distributions across groups. We compared proportions of respondents in each family member incarceration group identified as thriving, surviving, or suffering in their overall life evaluation. Similarly, we compared proportions of respondents identified with high well-being in each domain, across each family member incarceration history group. We used the Kruskal-Wallis test to assess trends in well-being across strata based on the number of immediate and extended family members incarcerated, and calculated estimates of life expectancy based on trends in the life evaluation score.^16^

To explore the possibility of these associations being driven by other covariates, we used 4 nested multivariate logistic regression models to estimate adjusted associations between family incarceration exposure and the odds of a thriving life evaluation. First, we accounted for the key sociodemographic characteristics of age, gender, race/ethnicity, and education level. Next, we adjusted for social and economic factors, including employment status, housing type, marital status, family size, and household income. In the third set of models, we adjusted for respondents’ addiction history. Finally, we adjusted for respondents’ own incarceration history. We estimated this set of models separately for immediate and extended family member incarceration exposure.

We then used logistic regression model outputs to calculate covariate-adjusted life expectancy estimates. First, we used model outputs for the adjusted probability of thriving from logistic regression models of family incarceration exposure and thriving as the outcome measure, adjusting for age, gender, race/ethnicity, education level, employment status, housing type, marital status, family size, household income, and addiction history. Next, we used an analogous set of models with suffering as the outcome measure to calculate model outputs for the adjusted probability of suffering. Finally, we used these adjusted probabilities of thriving and suffering to calculate adjusted Life Evaluation Index and life expectancy estimates. Statistical analyses excluded participants with missing data.

All statistical tests were 2-sided with α = .05. We conducted analyses in R statistical software version 4.0.0 (R Project for Statistical Computing) and weighted percentages using FamHIS-specified sampling weights to adjust the analytic sample to be representative of the US household adult population. The sampling weights account for recruitment into the baseline sample of 4041 respondents, stratified subsampling into the full FamHIS sample on the basis of family incarceration experience, and benchmarks the full survey sample of 2815 respondents to the US household adult population. Final regression model specifications were determined with tests for collinearity using a variance inflation factor cutoff of 2.0. Models were calculated using the Karlson-Holm-Breen method to allow for comparisons between sets of covariates.^24^ Data were analyzed from October 2019 to April 2020.

## Results

Among 2815 individuals included in this study, 1472 (51.7%) were women, 1765 (62.8%) were non-Hispanic White, and 868 (31.5%) were aged 35 to 54 years. A total of 1806 respondents (45.0%) reported having an immediate family member who was incarcerated, and 965 respondents (35.4%) reported having any extended family member incarceration, concordant with previous FamHIS estimates.^1^ Individuals with any family member who was incarcerated, compared with individuals with no family member who was incarcerated, were more likely to be Black (336 respondents [16.1%] vs 44 respondents [6.5%]; *P* < .001), live in lower-income households (eg, income <$24 999: 502 respondents [27.6%] vs 115 respondents [15.6%]; *P* < .001), have a history of drug or alcohol addiction (425 respondents [21.1%] vs 56 respondents [8.5%]; *P* < .001), or have a prior incarceration themselves (569 respondents [27.9%] vs 49 respondents [6.1%]; *P* < .001) (Table 1).

**Table 1.** Respondent Demographic Characteristics by Family Incarceration Experience

Characteristic	No. (weighted %)^a^			*P* value
	No family incarceration (n = 667)	Any family incarceration (n = 1989)	Overall (N = 2815)	
Age, y				
18-24	39 (10.5)	126 (10.8)	182 (11.2)	.01
25-34	116 (15.3)	497 (22.4)	651 (19.6)	
35-54	216 (33.4)	595 (29.5)	868 (31.5)	
55-64	108 (15.3)	399 (19.1)	530 (17.2)	
65-74	117 (15.7)	264 (12.9)	397 (13.8)	
≥75	71 (9.7)	108 (5.2)	187 (6.7)	
Gender				
Women	329 (49.1)	1070 (54.9)	1472 (51.7)	.047
Men	338 (50.9)	919 (45.1)	1343 (48.3)	
Race/ethnicity				
Black (non-Hispanic)	44 (6.5)	336 (16.1)	397 (11.9)	<.001
Hispanic	98 (17.4)	289 (16.4)	411 (16.2)	
White (non-Hispanic)	456 (65.6)	1211 (60.4)	1765 (62.8)	
Other (non-Hispanic)^b^	69 (10.5)	153 (7.1)	242 (9.1)	
Immediate family size, median (IQR), No.	6 (4-8)	7 (4-9)	6 (4-9)	<.001
Household income, $				
≤24 999	115 (15.6)	502 (27.6)	659 (23.4)	<.001
25 000-49 999	176 (26.3)	565 (27.1)	782 (26.2)	
50 000-74 999	113 (17.5)	358 (16.5)	503 (17.4)	
75 000-99 999	98 (15.1)	256 (12.7)	367 (13.3)	
≥100 000	165 (25.5)	308 (16.0)	504 (19.6)	
Housing type				
Single-family home	480 (74.7)	1352 (69.0)	1946 (71.6)	.09
Apartment	169 (21.8)	532 (25.3)	742 (23.6)	
Mobile home, trailer, boat, RV, or van	18 (3.5)	105 (5.7)	127 (4.8)	
Employment status				
Working	382 (56.8)	1184 (57.8)	1680 (58.2)	.59
Not working, seeking job	35 (6.3)	139 (8.2)	184 (7.9)	
Not working, not seeking job	198 (28.7)	524 (26.0)	751 (26.2)	
Not working, other	52 (8.2)	142 (7.9)	200 (7.6)	
Education				
<HS	32 (8.3)	151 (13.4)	188 (10.7)	<.001
HS graduate or GED	111 (23.8)	414 (30.5)	566 (28.7)	
Some college	234 (24.2)	929 (30.1)	1229 (27.7)	
≥Bachelor’s degree	290 (43.7)	495 (25.9)	832 (33.0)	
Marital status				
Never married	158 (25.0)	457 (24.0)	658 (25.1)	.51
Married or living with partner	374 (55.6)	1077 (53.9)	1538 (54.3)	
Widowed, divorced, or separated	135 (19.4)	455 (22.2)	619 (20.6)	
History of addiction				
Yes	56 (8.5)	425 (21.1)	497 (15.6)	<.001
No	593 (91.5)	1495 (78.9)	2216 (84.4)	
Own incarceration experience				
Yes	49 (6.1)	569 (27.9)	642 (18.8)	<.001
No	616 (93.9)	1415 (72.1)	2163 (81.2)	

Abbreviations: GED, general educational development; HS, high school; IQR, interquartile range; RV, recreational vehicle.

^a^ Proportions are weighted to be nationally representative of the US household population.

^b^ Includes American Indian, Alaskan Native, Asian, and multiracial respondents.

In unadjusted analyses, compared with those with no family member incarceration, individuals with any family member who was incarcerated had lower proportions of thriving in their life evaluation (69.5% [95% CI, 65.0%-75.0%] vs 56.9% [95% CI, 53.9%-59.9%]), and lower well-being in each domain (eg, physical thriving: 51.1% [95% CI, 46.2-56.0] vs 35.5% [95% CI, 32.6%-38.3%]) (Table 2). Compared with individuals with no family incarceration, individuals with an immediate family member who was incarcerated had lower odds of a thriving life evaluation (odds ratio [OR], 0.62; 95% CI, 0.52-0.71), as did individuals with any extended family member who was incarcerated (OR, 0.61; 95% CI, 0.52-0.71) (Table 3). The association between any immediate family member incarceration exposure and thriving life evaluation was attenuated but remained statistically significant after full covariate adjustment. The association between any extended family member incarceration and thriving life evaluation was no longer significant after adjustment for respondents’ own incarceration history and immediate family incarceration history (Table 3).

**Table 2.** Trends in Life Evaluation by Family Incarceration Experience

Family incarceration experience	Thriving, % (95% CI)^a^				
	Overall	Physical health	Mental health	Social well-being	Spiritual well-being
Overall (N = 2815)	63.3 (60.8-65.7)	41.7 (39.1-44.2)	57.4 (54.8-59.9)	34.2 (31.7-36.7)	63.4 (61.0-65.9)
No family incarceration (n = 667)	69.5 (65.0-75.0)	51.1 (46.2-56.0)	65.3 (60.6-70.0)	41.0 (36.1-45.9)	68.2 (63.6-72.7)
Any immediate family (n = 1806)	56.9 (53.9-59.9)	35.5 (32.6-38.3)	51.6 (48.7-54.6)	29.2 (26.5-31.8)	60.2 (57.3-63.1)
*P* value^b^	<.001	<.001	<.001	<.001	<.005
1 member	60.2 (55.9-64.5)	40.2 (36.0-44.4)	54.5 (50.2-58.8)	34.0 (30.0-38.1)	63.6 (59.3-67.8)
2-3 members	57.3 (52.4-62.3)	35.3 (30.5-40.1)	51.2 (46.2-56.2)	27.0 (22.6-31.4)	59.3 (54.1-68.7)
>3 members	47.7 (40.6-54.8)	23.6 (17.4-29.8)	45.1 (38.0-52.2)	20.7 (14.9-26.5)	53.4 (46.2-60.6)
*P* value for trend	<.001	<.001	<.001	<.001	<.05
Any extended family (n = 965)	57.7 (53.5-62.0)	34.2 (30.2-38.2)	54.5 (50.2-58.7)	27.0 (23.2-30.8)	59.5 (55.3-63.6)
*P* value^b^	<.001	<.001	<.001	<.001	<.01
1 member	67.9 (59.8-76.0)	40.7 (32.0-49.4)	64.4 (56.2-72.6)	36.1 (27.6-44.5)	68.8 (60.2-77.4)
2-3 members	61.3 (53.6-69.0)	37.9 (30.6-45.3)	57.0 (49.3-64.7)	28.8 (21.6-36.0)	61.4 (54.1-68.7)
>3 members	49.3 (43.0-55.6)	27.8 (22.4-33.2)	47.2 (41.0-53.4)	20.7 (15.8-25.6)	52.9 (46.7-59.2)
*P* value for trend	.006	.06	.006	<.001	<.001
Both extended and immediate family (n = 782)	53.8 (49.2-58.3)	32.5 (28.3-36.6)	49.8 (45.3-54.3)	24.2 (20.4-28.0)	55.3 (50.8-59.8)

^a^ Proportions are weighted to be nationally representative of the US household population. Number and percentage missing by well-being variable: life evaluation, 60 respondents (2.1%); physical health, 4 respondents (0.1%); mental health, 7 respondents (0.3%); social well-being, 31 respondents (1.1%); spiritual well-being, 12 respondents (0.4%). Number and percentage of missing by family incarceration variable: any family incarceration, 159 respondents (5.7%); any immediate family incarceration, 7 respondents (0.3%); any extended family incarceration, 498 respondents (17.6%).

^b^ *P* value for χ^2^ comparison against those with no family incarceration.

**Table 3.** Adjusted Associations Between Family Incarceration Experience and Life Evaluation

Model^a^	Family incarceration experience, OR (95% CI)	
	None	Any
Immediate family incarceration		
Thriving, No.	654	1004
Not Thriving, No.	318	773
Model 1A	1 [Reference]	0.62 (0.52-0.71)
Model 2A	1 [Reference]	0.70 (0.59-0.83)
Model 3A	1 [Reference]	0.69 (0.58-0.81)
Model 4A	1 [Reference]	0.69 (0.58-0.82)
Model 5A	1 [Reference]	0.72 (0.58-0.89)
Extended family incarceration		
Thriving, No.	843	528
Not Thriving, No.	467	427
Model 1B	1 [Reference]	0.61 (0.52-0.71)
Model 2B	1 [Reference]	0.71 (0.59-0.86)
Model 3B	1 [Reference]	0.75 (0.62-0.91)
Model 4B	1 [Reference]	0.75 (0.62-0.92)
Model 5B	1 [Reference]	0.84 (0.68-1.03)

Abbreviation: OR, odds ratio.

^a^ Model 1A is the unadjusted association of immediate family incarceration with life evaluation. Model 2A adjusted for model 1A plus age, gender, race/ethnicity, and education level. Model 3A adjusted for model 2A plus household income, home type, employment status, marital status, and family size. Model 4A adjusted for model 3A plus history of addiction. Model 5A adjusted for model 4A plus own incarceration history and extended family incarceration. Model 1B is the unadjusted association of extended family incarceration with life evaluation. Model 2B adjusted for model 1B plus age, gender, race/ethnicity, and education level. Model 3B adjusted for model 2B plus household income, home type, employment status, marital status, family size. Model 4B adjusted for model 3B plus history of addiction. Model 5B adjusted for model 4B plus own incarceration history and immediate family incarceration. Sample sizes are unweighted. Regressions are weighted to be nationally representative of the US household population, and use the Karlson-Holm-Breen method to allow for model comparisons. Owing to missingness in the data for immediate family incarceration, extended family incarceration, and life evaluation, cell counts do not sum to 2815.

Individuals with greater numbers of immediate or extended family members ever incarcerated were progressively less likely to report thriving life evaluations and lower levels of well-being in each domain, with the exception of individuals with extended family members incarcerated in the domain of physical health (Table 2). Compared with individuals without family member incarceration, individuals with any immediate family member incarceration had a 12.5-point lower Life Evaluation Index, equivalent to an estimated mean (SE) of 2.6 (0.03) fewer years of life expectancy, and individuals with 3 or more immediate family members ever incarcerated had a lower life evaluation equivalent to an estimated mean (SE) of 4.6 (0.07) fewer years of life expectancy (Table 4).

**Table 4.** Trends in Life Expectancy by Family Incarceration Experience^a^

Family incarceration experience	No.	Life Evaluation Index score (SE)	% (95% CI)		Change in life expectancy, mean (SE), y^b^	
			Thriving life evaluation	Suffering life evaluation	Unadjusted	Adjusted
Overall	2755	60.6 (2.2)	63.3 (60.8 to 65.7)	2.7 (1.8 to 3.6)	NA	NA
None	643	66.9 (4.5)	69.5 (65.0-75.0)	2.6 (0.8 to 4.4)	NA	NA
Any immediate family incarceration	1777	54.4 (2.8)	56.9 (53.9 to 59.9)	2.5 (1.7 to 3.3)	−3.56 (0.03)	−2.60 (0.03)
1 member	844	58.2 (4.0)	60.2 (55.9 to 64.5)	2.0 (1.0 to 3.1)	−2.48 (0.04)	−1.31 (0.04)
2-3 members	614	54.6 (4.8)	57.3 (52.4 to 62.3)	2.7 (1.2 to 4.2)	−3.51 (0.05)	−3.02 (0.05)
>3 members	319	44.6 (6.8)	47.7 (40.6 to 54.8)	3.1 (1.3 to 5.0)	−6.36 (0.07)	−4.62 (0.07)
Any extended family incarceration	998	54.2 (3.7)	57.7 (53.5 to 62.0)	3.2 (1.5 to 5.1)	−3.62 (0.04)	−2.17 (0.04)
1 member	198	65.6 (8.1)	67.9 (59.8 to 76.0)	2.4 (0.0 to 5.0)	−0.37 (0.08)	−1.45 (0.09)
2-3 members	325	55.8 (6.4)	61.3 (53.6 to 69.0)	5.5 (0.8 to 10.3)	−3.17 (0.06)	−3.74 (0.07)
>3 members	432	47.4 (5.9)	49.3 (43.0 to 55.6)	1.9 (0.7 to 3.3)	−5.56 (0.06)	−4.02 (0.06)
Duration of longest family member incarceration						
<1 y	1239	56.8 (3.3)	58.9 (55.3 to 62.4)	2.1 (1.3 to 3.0)	−2.88 (0.03)	−1.03 (0.03)
1-5 y	324	50.1 (6.7)	52.6 (45.5 to 59.6)	2.5 (0.7 to 4.2)	−4.79 (0.07)	−2.37 (0.07)
6-10 y	102	55.3 (11.4)	60.9 (49.1 to 72.7)	5.6 (0 to 12.1)	−3.31 (0.11)	−4.45 (0.12)
>10 y	110	42.8 (11.7)	46.2 (33.6 to 58.8)	3.4 (0 to 7.4)	−6.87 (0.12)	−5.05 (0.12)

Abbreviation: NA, not applicable.

^a^ Sample sizes are unweighted. Proportions and life expectancy estimates are weighted to be nationally representative of the US household population. Owing to missingness in the data for life evaluation and family member incarceration characteristics, cell counts do not sum to 2815.

^b^ Change in life expectancy is relative to those with no family incarceration. Adjusted change in life expectancy is calculated using regression model outputs adjusting for age, gender, race/ethnicity, education level, household income, home type, employment status, marital status, family size, and history of addiction.

A total 62.9% (95% CI, 55.8%-70.0%) of Black respondents had an immediate family member who was incarcerated, and 52.6% (95% CI, 95% CI, 45.1%-60.2%) of Black respondents had an extended family member who was incarcerated, compared with 42.5% (95% CI, 95% CI, 39.6%-45.5%) of White respondents with an immediate family member who was incarcerated (*P* < .001) and 32.0% (95% CI, 28.9%-35.1%) of White respondents with an extended family member who was incarcerated (*P* < .001). Black respondents had progressively higher proportions of family members with longer durations of incarceration compared with White respondents (eTable 2 in the Supplement). Among Black respondents, 11.9% (95% CI, 7.6%-16.2%) had a family member incarcerated for 10 or more years, compared with only 1.4% (95% CI, 0.8%-2.0%) of White respondents. Almost one-quarter of Black respondents (23.8% [95% CI, 18.5%-29.1%]) have had more than 3 immediate family members incarcerated, compared with 5.2% (95% CI, 4.1%-6.3%) of White respondents. Among respondents with any family member incarceration, Black respondents had an estimated mean (SE) of 0.46 (0.04) fewer years of life expectancy compared with White respondents.

## Discussion

In this cross-sectional study using data from a nationally representative survey, we found that any family member incarceration was associated with lower well-being and 2.6 fewer years of life expectancy, similar to the association with being personally incarcerated.^25^ Having 3 or more immediate family members incarcerated was associated with a progressively worse life evaluation, an association equivalent to 4.6 fewer years of life expectancy. This disparity in life expectancy is comparable to having a myocardial infarction.^26^ These findings underscore the far-reaching impact of mass incarceration on both individuals who are incarcerated^27^ and their family members. With more than 60% of the US population ever having any family member incarcerated, and an even higher prevalence among Black individuals,^1^ our findings suggest that policy reforms that decrease incarceration could have wide-reaching outcomes for population-level and racial/ethnic disparities in health and mortality.

Our findings highlight the multidimensional associations of incarceration of any family member with overall life evaluation, physical health, mental health, and social, financial, and spiritual well-being. Immediate family member incarceration was associated with larger decreases in projected life expectancy compared with extended family member incarceration. Furthermore, the association between extended family member incarceration and well-being was not statistically significant after adjusting for all covariates, suggesting that extended family member incarceration may not be associated with well-being to the same extent as immediate family member incarceration.

The findings of this study add to an evolving body of literature that has identified several health impacts of family member incarceration, including increased risk of chronic diseases^2,8,10,11^ and adverse mental health outcomes.^3,7,13^ For example, a study by Lee et al^10^ found that women with an incarcerated family member had a higher risk of obesity and cardiovascular disease, potentially due to increased stress and social demands with concurrently reduced material support, which could impede self-care behaviors. Similarly, a study by Green et al^13^ found that having an incarcerated adult son was associated with increased maternal psychological distress and burden of caregiving and financial support for grandchildren.

Our findings also corroborate work highlighting the negative social and financial outcomes associated with family member incarceration. Existing literature has closely examined how community stigma around incarceration can reduce social support for family members of incarcerated individuals,^6,7,8^ in addition to the economic impacts of family member incarceration. Conviction-related costs have a mean of approximately $13 000, and these costs are typically paid by family members on top of indirect financial consequences, such as time off from work for visitation.^28^ The financial burden from incarceration of the individual providing a family’s primary source of income can drive already economically unstable family members into larger amounts of debt and housing instability.^5^

Additionally, we identified large racial differences in family incarceration experience. Compared with White respondents, Black respondents were 2.4-fold more likely to have multiple immediate family members incarcerated, and 8.5-fold more likely to have an immediate family member incarcerated for a decade or longer. These findings suggest that differences in family incarceration may be an important factor in shaping Black-White disparities in national well-being, adding further evidence of the disproportionate impact of mass incarceration on the well-being of Black families and its intergenerational effects.^2,3,4^

Our study design was strengthened by a large, nationally representative study sample with high-quality sampling methods and low levels of missing data. Our analyses used detailed contextual data within the FamHIS, including family relationship structure, family member incarceration experiences, and respondent incarceration experiences. Our findings are compelling with their associations, dose-dependent gradients with degree of family member incarceration exposure, and consistency across all measures of well-being.

### Limitations

This study has some limitations. The FamHIS data are susceptible to recall and social desirability bias, challenges faced by many key data sources on incarceration and its associations with health.^29^ These findings are limited by the self-reported and cross-sectional nature of the data; we cannot elucidate the temporality nor causality of family member incarceration exposures and well-being.

Furthermore, community-level factors, like neighborhood policing presence, housing quality, or proximity to grocery stores, may influence well-being and were not included in the FamHIS, although our adjustment for race/ethnicity as a proxy for structural racism and adjustment for household income, education, and housing type may account for some aspects of community-level factors. Although the FamHIS allows inference to the population of all US noninstitutionalized adults, this address-based panel excludes individuals experiencing homelessness or who were institutionalized at the time of data collection. This is a challenge of research on the consequences of incarceration exposure, because no nationally representative data capturing well-being and including these groups are currently available, to our knowledge.^30^

## Conclusions

The findings of this cross-sectional study suggest that family member incarceration was associated with lower well-being and lower projected life expectancy, with evidence of a disproportionate burden among Black family members. Our findings suggest that family member well-being may be an important avenue through which incarceration is associated with racial health disparities. Ongoing decarceration efforts could have wide-ranging outcomes in population-level well-being, life expectancy, and racial/ethnic disparities by minimizing detrimental effects of incarceration on individuals who are incarcerated and on their nonincarcerated family members.

## Notes

Supplement. **eAppendix 1.** Methodological Overview of the Sampling Design of the Family History of Incarceration Survey (FamHIS) **eAppendix 2.** Further Details on Well-being Measure and Results by Well-being Domain **eFigure.** Cantril’s Self-Anchoring Scale, an Assessment of Life Evaluation and a Measure of Well-being **eTable 1.** 100 Million Healthier Lives Adult Well-being Assessment Scoring System **eTable 2.** Family Member Incarceration Characteristics by Race
