# Supplementary material for: Exposure to Family Member Incarceration and Adult Well-being in the United States
Source: JAMA Netw Open. 2021 May 28;4(5):e2111821. doi: 10.1001/jamanetworkopen.2021.11821 (PMC8164096; doi:10.1001/jamanetworkopen.2021.11821)
Supplement: Supplement. — eAppendix 1. Methodological Overview of the Sampling Design of the Family History of Incarceration Survey (FamHIS) eAppendix 2. Further Details on Well-being Measure and Results by Well-being Domain eFigure. Cantril’s Self-Anchoring Scale, an Assessment of Life Evaluation and a Measure of Well-being eTable 1. 100 Million Healthier Lives Adult Well-being Assessment Scoring System eTable 2. Family Member Incarceration Characteristics by Race [file jamanetwopen-e2111821-s001.pdf]

Supplemental Online Content

Sundaresh R, Yi Y, Harvey TD, et al. Exposure to family member incarceration and adult well-being in the United States. *JAMA Netw Open.* 2021;4(5):e2111821. doi:10.1001/jamanetworkopen.2021.11821

eAppendix 1. Methodological Overview of the Sampling Design of the Family History of Incarceration Survey (FamHIS)

eAppendix 2. Further Details on Well-being Measure and Results by Well-being Domain

eFigure. Cantril's Self-Anchoring Scale, an Assessment of Life Evaluation and a Measure of Well-being

eTable 1. 100 Million Healthier Lives Adult Well-being Assessment Scoring System

eTable 2. Family Member Incarceration Characteristics by Race

This supplemental material has been provided by the authors to give readers additional information about their work.

© 2021 Sundaresh R et al. *JAMA Network Open.*

eAppendix 1. Methodological Overview of the Sampling Design of the Family History of Incarceration Survey (FamHIS)

This study uses data from the Family History of Incarceration Survey (FamHIS), a nationally representative cross-sectional study about personal and family incarceration experience. These data were collected between August – September 2018, and were originally designed to estimate the prevalence of incarceration experience among family members. The baseline sample of FamHIS respondents includes 4,041 adults that were recruited by the National Opinion Research Center (NORC) using a stratified sampling design. This study's analysis is based on the 2,815 respondents who participated in the full FamHIS questionnaire. Both the baseline FamHIS sample (N=4,041) and the subsample of FamHIS respondents who participated in the full survey questionnaire (N=2,815) were designed to be representative of the non-institutionalized U.S. household population over the age of 18.

The AmeriSpeak Panel

The FamHIS sample was drawn from NORC's AmeriSpeak panel of approximately 30,000 households, a probability-based panel designed to be representative of the U.S. household adult population. AmeriSpeak provides sample coverage of over 97% of the U.S. household population and includes oversampling of 18-24 year-olds, persons of Hispanic ethnicity, and persons identifying as non-Hispanic African Americans, to ensure adequate coverage of these groups, for statistical analyses. Initial recruitment to the AmeriSpeak panel was conducted using multiple strategies to maximize recruitment outcomes: mail, telephone, and in-person interviewers. For follow-up to initially non-responsive sampled households, NORC employed a two-stage recruitment strategy, beginning with mail and phone contact and modest incentives, and then using additional mailings, enhanced incentives, and in-person recruitment by field staff.

The FamHIS Sample

AmeriSpeak panelists participate in studies conducted by NORC or studies conducted by NORC clients. The FamHIS is part of the latter category, and was conducted by a research team from Cornell University. Based on pretesting and power analyses, the FamHIS research team and NORC determined initial target sizes for a study of family incarceration, with a target to recruit 1,000 respondents without any family incarceration and 1,750 respondents with any family incarceration experience into the FamHIS sample. Using these targets and internal estimates of anticipated non-response across different groups, NORC invited a carefully designed internal sample of 11,992 AmeriSpeak panelists that was optimized to yield these targets while remaining representative of the U.S. household adult population (described below). This internal sample of panelists was offered a screening tool about family incarceration experience, and of these 11,992 panelists, 4,041 completed the screening tool and were eligible for participation in the full FamHIS questionnaire. This set of 4,041 screened panelists constitutes the baseline FamHIS sample, of which 1,009 respondents without any family incarceration and 1,806 respondents with any family incarceration participated in the full survey questionnaire. This final set of 2,815 (1,009 + 1,806) respondents closely aligns with the original targets and constitutes the full FamHIS sample. This full questionnaire includes measures of family incarceration experience, own criminal legal system experience, and measures of wellbeing.

Panelists were offered an incentive equivalent of $4 for completing the FamHIS questionnaire, which was increased to $10 in the last two days of the recruitment window in order to boost cooperation. Recruitment materials included a study brochure and a summary of the privacy

© 2021 Sundaresh R et al. *JAMA Network Open.*

policy. The study questionnaire was approved by Institutional Review Board of Cornell University. The study questionnaire was offered in both English and Spanish and was offered in both web and phone formats to maximize response.

Sample Weights

The internal sample of 11,992 panelists was designed to be representative of the U.S. household adult population by using stratified sampling from the AmeriSpeak panel on the basis of age, race/ethnicity, education, and gender, based on national distributions of these data. This internal sample was derived from AmeriSpeak panel, which is itself nationally representative on the basis of census tract, age, and race/ethnicity, with additional measures (described above) to adequately cover difficult-to-reach groups.

The FamHIS data include two sets of final sampling weights, which factor these sampling methods to adjust the baseline FamHIS sample (N=4,041) and the final FamHIS sample (N=2,815) to remain representative of the U.S. household adult population as well. WEIGHT1 includes all the baseline sample (N=4,041) and weights the screening data to the U.S. household adult population. A second set of weights, WEIGHT2 was created for *only* the respondents who participated in the full survey (N=2,815) and also weights the full survey questionnaire—including items on own CLS involvement and family incarceration exposure, and wellbeing-- to the U.S. household adult population. Both WEIGHT1 and WEIGHT2 account for the sampling design of the AmeriSpeak panel, stratified recruitment into the internal sample of panelists invited to participate in the FamHIS, voluntary entry into the baseline FamHIS sample by completion of the screener, and adjustment for initial non-response and non-response to the first stage of follow-up recruitment. WEIGHT2 was adjusted further using an iterative raking strategy to account for subsampling of panelists from the baseline FamHIS sample into the rest of the survey, using benchmark population totals and distribution with respect to age, sex, education, race/ethnicity, and Census Division, using population estimates from the Current Population Survey as reference. The 1,009 individuals without any family incarceration in the full FamHIS sample was a random subsample of 2,231 individuals who reported having no family incarceration within the baseline sample of 4,041, and this subsampling is also factored into the design of WEIGHT2. The analyses in this study are weighted using WEIGHT2.

© 2021 Sundaresh R et al. *JAMA Network Open.*

eAppendix 2. Further Details on Well-being Measure and Results by Well-being Domain

100 Million Healthier Lives Adult Wellbeing Assessment

1) Please imagine a ladder with steps numbered from zero at the bottom to ten at the top. The top of the ladder represents the best possible life for you and the bottom of the ladder represents the worse possible life for you.

Indicate where on the ladder you feel you personally stand right now.

2) On which step do you think you will stand about five years from now?

3) Now imagine the top of the ladder represents the best possible *financial situation* for you, and the bottom of the ladder represents the worst possible *financial situation* for you. Please indicate where on the ladder you stand right now.

4) In general, how would you rate your physical health?
Excellent Very Good Good Fair Poor

5) In general, how would you rate your mental health, including your mood and your ability to think?
Excellent Very Good Good Fair Poor

6) How often do you get the social and emotional support you need?
Always Usually Sometimes Rarely Never

7) How strongly do you agree with this statement? "I lead a purposeful and meaningful life."
Strongly Agree Agree Slightly Agree Neither Agree nor Disagree Disagree Strongly Disagree

Reproduced with permission from Stiefel MC, Riley CL, Roy B, Ramaswamy R, Stout S. 100 Million Healthier Lives Measurement System: Progress to Date. 2016. *100 Million Healthier Lives Metrics Development Team*. Available at https://www.100mlives.org/wp-content/uploads/2016/03/FINAL_100MHL-Measurement-Framework-Report_2016-03-17.pdf. Accessed April 10, 2019.

© 2021 Sundaresh R et al. *JAMA Network Open.*

eFigure. Cantril's Self-Anchoring Scale, an Assessment of Life Evaluation and a Measure of Well-being

Reproduced with permission from Stiefel MC, Riley CL, Roy B, Ramaswamy R, Stout S. 100 Million Healthier Lives Measurement System: Progress to Date. 2016. *100 Million Healthier Lives Metrics Development Team*. Available at https://www.100mlives.org/wp-content/uploads/2016/03/FINAL_100MHL-Measurement-Framework-Report_2016-03-17.pdf. Accessed April 10, 2019.

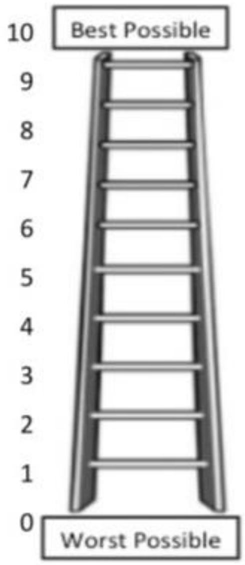

1) Indicate where on the ladder you feel you personally stand right now. (Current Life Satisfaction)
2) On which step do you think you will stand about five years from now? (Future Life Optimism)

© 2021 Sundaresh R et al. *JAMA Network Open.*

eTable 1. 100 Million Healthier Lives Adult Well-being Assessment Scoring System

**Wellbeing Domain**	**Thriving**	**Surviving**	**Suffering**
Life Evaluation			
Current outlook (0 – 10)	7+	5-6	0-4
5-year outlook (0 – 10)	8+	5-7	0-4
Life evaluation overall	Both thriving		Both suffering
Physical health (1-5)	Excellent (5)/ Very good (4)	Good (3)	Fair (2) / Poor (1)
Mental health (1-5)	Excellent (5)/ Very good (4)	Good (3)	Fair (2) / Poor (1)
Spiritual wellbeing (1-7)	Strongly agree (7)/ Agree (6)	Slightly agree (5)/ Neither agree nor disagree (4)/ Slightly disagree (3)	Disagree (2)/ Strongly disagree (1)
Social wellbeing			
Financial situation (0 – 10)	7+	5-6	0-4
Social support (1-5)	Always (5)/ Usually (4)	Sometimes (3)	Rarely (2)/ Never (1)
Social wellbeing overall	Both thriving		Both suffering

Reproduced with permission from Stiefel MC, Riley CL, Roy B, Ramaswamy R, Stout S. 100 Million Healthier Lives Measurement System: Progress to Date. 2016. *100 Million Healthier Lives Metrics Development Team*. Available at https://www.100mlives.org/wp-content/uploads/2016/03/FINAL_100MHL-Measurement-Framework-Report_2016-03-17.pdf. Accessed April 10, 2019.

© 2021 Sundaresh R et al. *JAMA Network Open.*

eTable 2. Family Member Incarceration Characteristics by Race

**Family incarceration characteristics**	**Black (%)**	**White (%)**	**P-value**
Longest duration	**N = 396**	**N = 1760**	<0.001
0 days	37.1	57.5	
1 day	10.5	12.4	
2 days - 1 month	10.2	11.7	
1 month - 1 year	11.1	8.1	
1-5 years	14.3	6.9	
6-10 years	4.9	2.0	
> 10 years	11.9	1.4	
#Immediate family members incarcerated	**N = 397**	**N = 1765**	<0.001
0	37.1	57.6	
1	16.8	22.9	
2-3	22.4	14.3	
>3	23.8	5.2	

© 2021 Sundaresh R et al. *JAMA Network Open.*
